# miR-4443 Participates in the Malignancy of Breast Cancer

**DOI:** 10.1371/journal.pone.0160780

**Published:** 2016-08-09

**Authors:** Xiu Chen, Shan-liang Zhong, Peng Lu, Dan-dan Wang, Si-ying Zhou, Su-jin Yang, Hong-yu Shen, Lei Zhang, Xiao-hui Zhang, Jian-hua Zhao, Jin-hai Tang

**Affiliations:** 1 The Fourth Clinical School of Nanjing Medical University, Nanjing, China; 2 Department of General Surgery, Jiangsu Cancer Hospital Affiliated to Nanjing Medical University, Nanjing, China; 3 Center of Clinical Laboratory Science, Jiangsu Cancer Hospital Affiliated to Nanjing Medical University, Nanjing, China; 4 School of Public Health Nanjing Medical University, Nanjing, China; 5 Department of General Surgery, First Affiliated Hospital of Nanjing Medical University, Nanjing, China; Wayne State University, UNITED STATES

## Abstract

**Purpose:**

Chemo-resistance is the leading cause of failure in cancer therapy, however, much remains to be understood about the intrinsic mechanisms. In the present study, we discovered the novel miR-4443 that regulated malignancy of breast cancer both *in vitro* and *in vivo*.

**Methods:**

We examined the expression of miR-4443 in MDA-MB-231/S and MDA-MB-231 Epirubicin-resistant cell lines with 76 breast cancer formalin-fixed paraffin-embedded tissues by real-time PCR. Also, we investigated the loss- and gain-functions of miR-4443 by MTT assay and flow cytometry. Furthermore, we detected miR-4443 mediated tissue inhibitor of metalloproteinase 2 expression in cells by TargetScan, RT-qPCR and western blot.

**Results:**

We identified the up-regulated expression of miR-4443 in Epi-resistant cell lines versus MDA-MB-231/S cell(Epi versus S) and in post-chemotherapy FFPE tissues, along with statistically differential expressions in PR(partial response) versus SD(stable disease)/PD(progressive disease) patients. Overexpression of miR-4443 increased the IC50 value of Epi for the target cells transfected, while inhibition of miR-4443 could restored sensitivity of the target cells to Epi. Besides, down-regulation of endogenous miR-4443 by miRNA-inhibitors significantly enhanced Epi-induced apoptosis while up-regulation of miR-4443 by miRNA-mimics lead to less Epi-induced apoptotic cells. Consequently, changes in TIMP2 mRNA and protein expression revealed that miR-4443 mimics suppressed expression of TIMP2 and induced migration in breast cancer cells. Furthermore, TIMP2 expression associated with better prognosis(HR = 0.721, 95%CI: 0.529–0.983).

**Conclusions:**

We revealed that miR-4443 induced malignancy of breast cancer mainly in chemo-resistance aspect for the very first time, providing a novel biomarker in breast cancer diagnosis and therapy.

## Introduction

Despite of the advanced development of early diagnosis and appropriate treatment in breast cancer patients[1–5], chemo-resistance[6] seems to be the crucial issue that brings about the high recurrence rate and poor prognosis in breast cancer patients. Therefore, an effective detection of informative biomarkers to challenge the drug resistance is in desperate demand, which ultimately contributes to the promoted prognosis of breast cancer patients[7].

MiR-4443 was first detected in 2015 by Xun et al.[8], but rare evidence had confirmed the association of miR-4443 and cancerous malignancy. Since miRNAs participated in a mass of varied biological processes, including cell cycle, cell proliferation, apoptosis, chemo-resistance and metabolic processes [9–14], we studied the role of miR-4443 in breast cancer malignancy mainly through drug resistance.

Tissue inhibitor of metalloproteinase(TIMP) family have the ability to directly suppress the proliferation of endothelial cells, in addition to its inhibitory role against metalloproteinases. So we further assessed TIMP2 mRNA and protein expressions in MDA-MB-231 breast cancer cells affected by abnormal expression of miR-4443.

In this study, we analyzed that miR-4443 induced the malignancy of breast cancer mainly in chemo-resistance, providing a novel biomarker in breast cancer diagnosis and therapy.

## Materials and Methods

### Ethical statement

All procedures performed in studies involving human participants were in accordance with the ethical standards of the guidelines of ethics committee of Nanjing Drum Tower Hospital and with the 1964 Helsinki declaration and its later amendments or comparable ethical standards. Also, written informed consent was obtained from all patients.

### Cell lines

Human breast cancer cell lines MDA-MB-231, used in this study, were obtained from the Cell Bank of the Chinese Academy of Sciences (Shanghai, China). Epirubicin(Epi) resistant subline (MDA-MB-231/Epi) was established by exposing to Epi from 10 nM to 800 nM *in vitro* in our laboratory. Parental MDA-MB-231 cultured synchronously in the absence of drug was used as a control (called MDA-MB-231/S). The IC50 (inhibitory concentration to produce 50% cell death) values of Epi were 10.39 and 0.16 μM for MDA-MB-231/Epi and MDA-MB-231/S cells, respectively.

MDA-MB-231 cells were cultured in Dulbecco’s modified Eagle’s medium (DMEM) high glucose (HyClone), supplemented with 10% fetal bovine serum (FBS), 80 U/ml penicillin with 800 ug/ml streptomycin or 100 U/ml penicillin with 100 ug/ml streptomycin and incubated at 37°C and 5% CO2 in a humidified chamber atmosphere. The cell lines were cultured in drug free medium for two weeks before subsequent experiments to avoid the influence of drug.

### Total RNA extraction, Reverse transcription and quantitative Real-time PCR of cells

Total RNA was extracted using RNAsimple Total RNA kit (TIANGEN BIOTECH, Beijing, China) according to the manufacturer’s instructions. The concentration and quality of the RNA were measured by the UV absorbance at 260 and 280 nm (260/280 nm) on Nanodrop 2000 spectrophotometry (Thermo Scientific, USA).

Expression of miR-4443 was analyzed by using MiR-X miRNA qRT-PCR SYBR Kit (638314; Clontech Laboratories, USA) according to the manufacturer instructions on Roche LightCycler 480 II. 5ul mRQ Buffer (2x), 3.75ul RNA sample (0.25–8μg) and mRQ Enzyme 1.25ul were mixed in an RNase-free 0.2 ml tube and incubated for 1 hour at 37°C, then terminated at 85°C for 5min to inactivate the enzymes, later 90μl ddH2O was added to prepare for quantification protocols. Real-time quantitative PCR (RQ-PCR) was performed in a final volume of 25ul, containing 2ul of the cDNA template, 9ul ddH2O, 12.5ul SYBR Advantage Premix, 0.5ul ROX Dye, 0.5ul mRQ 3’ Primer and 0.5ul miRNA-specific Primer: TTGGAGGCGTGGGTTTT(miR-4443). The thermal profile for qRT-PCR was 95°C for 30 sec followed by 40 cycles of 95°C for 5 sec, 60°Cfor 20 sec, followed by melting curve detection. U6 snRNA was used as internal control to normalize miRNA expression in cells and tissues. Quantitation of tissue inhibitor of metalloproteinase 2(TIMP2) mRNA was performed by using Bu-SuperScript RT Kit (Biouniquer Technology, Nanjing, China) and SYBR Premix Ex Taq system (Roche, Australia) with forward primer as 5’-AGTGGACTCTGGAAACGACA-3’ and reverse prime as 5’-CGGCCTTTCCTGCAATGAGA-3’(TIMP2). β-actin was used as the endogenous control. The primers for β-actin were 5’-CACCTTCTACAATGAGCTGCGTGTG-3’ and 5’-ATAGCACAGCCTGGATAGCAACGTAC-3’. The Ct values for each gene were normalized to endogenous control, and the relative fold change values were calculated by using the ΔΔCt method in triplicates.

### Breast cancer tissues

A retrospective search was conducted through the computerized database at the Department of Pathology in Nanjing Drum Tower Hospital, for diagnosed breast cancer cases from January 2010 to February 2015. After comprehensive skimming, we selected 49 breast cancer cases with therapeutic response assessments of neoadjuvant chemotherapy such as PR(partial response), SD(stable disease) and PD(progressive disease) based on Response Evaluation Criteria in Solid Tumors Group (RECIST)[14]. Additionally, the diagnosis and classification of breast cancer patients depended on the Tumor-Node-Metastasis (TNM) system of American Joint Committee on Cancer (AJCC) [15].

Finally, we retrieved 76 breast tumor formalin-fixed paraffin-embedded(FFPE) blocks consistent with the study criteria. The blocks were from 27 needle biopsy tissues before treatment and 27 postoperative tissues after neoadjuvant chemotherapy in 27 patients, with 22 available postoperative tissues after neoadjuvant chemotherapy in the other 22 patients, amounting to 76 FFPE blocks. The samples were incubated for 5 to 10 hours in 10% neutral-buffered formalin before being alcohol-dehydrated and embedded in paraffin, and were stored at room temperature until use. The proportion of tumor cells in each sample was above 30%, as verified on a hematoxylin and eosin(HE) stained serial section by the same pathologist. The study protocol was approved by the guidelines of ethics committee of Nanjing Drum Tower Hospital and the 1964 Helsinki declaration and its later amendments or comparable ethical standards, and was reviewed and approved by the Nanjing Medical University ethics committee. Written informed consent was obtained from all patients. None of the authors were the attending physicians for any of the patients whose tissue samples were collected, and no one had access to potentially identifying patient information.

### Isolation miRNA from formalin-fixed paraffin-embedded tissues

Total RNA was extracted from tumorous breast tissues using RecoverAll™ Total Nucleic Acid Isolation Kit (Ambion, Carlsbad, CA, USA) as the manufacturer’s protocol. FFPE tissue blocks were cut into 10μm slices using a microtome following placed on slides and every 3 slides endured deparaffinizion by immersing in 100% xylene for 30min, and hydration through graded ethanols(100%, 85%, 75%) for 15min each. Next, tumor cell sections were scraped into 1.5ml centrifuge tubes according to the standard of HE staining to eliminate the influence of normal cells. Furthermore, the proteins were degraded with 100 μL digestion buffer and 4 μL protease, followed by incubation for 15 minutes at 50°C and for 15 minutes at 80°C. Subsequently, RNA was isolated by adding 395 μL of buffer containing 120 μL Isolation Additive and 275 μL absolute ethanol, along with passage through a purification column. The column was then washed twice with Wash 1 and Wash 2/3 from the kit, and DNase treatment was performed, followed by three additional washings. Finally, RNA was eluted in 60 μL of Elution Solution from the kit at room temperature (RT) according to the manufacturer’s instructions. After purification, the integrity and concentration of total RNA samples was measured through the absorbance at 260 and 280 nm with the Nanodrop 2000 spectrophotometry (Thermo Scientific, USA). RT-qPCR was conducted using the method described above.

### Transfection experiment

Cells in logarithmic phase were collected with a confluence of 80–90%, prior to transfection performed using a Nepa21 pulse generator (Nepa Gene, Chiba, Japan). Briefly, 10^6^ cells in 100ul antibiotics-free DMEM mixed with miR-4443-inhibitors(5’-AAAACCCACGCCUCCAA-3’) or miR-4443 mimics(5’-UUGGAGGCGUGGGUUU-3’)(GenePharma Co., Ltd, Shanghai, China) at a concentration of 40nM were added into an electrode champer. The parameters of NEPA 21 electroporator were as follows: voltage, 125V; pulse length, 5ms; pulse interval, 5ms; number of pulses, 2; decay rate, 10%; polarity + as poring pulse and voltage, 20V; pulse length, 50ms; pulse interval, 50ms; number of pulses, 5; decay rate 40%; polarity +/- as transfer pulse. Cells transfected with negative control miRNA mimics (mimics-NC) or negative control miRNA inhibitors (inhibitors-NC) were used as negative controls and cells transfected without external genes were used as blank controls. After transfection, the cells of each electrode chamber were plated into six-well or 96-well plates with non-antibiotics-culture medium and FBS for 24 hours.

### MTT assay

After transfection, the 100μL cells were seeded in 96-well plates at a density of 8×10^4^/ml for 24 h, following by various concentrations of Epi added to the cells(quadruplicate wells per condition). Two days later, 20μl of 5 mg/ml 3-(4,5-dimethylthiazol-2-yl)-2,5-diphenyltetrazolium bromide(MTT)(Sigma, Germany) was added to each well, and the plates were incubated for another 4 h. After removal of the culture medium, the cells in each well were mixed with 150μl of dimethyl sulfoxide (DMSO, AMRESCO, America) and the absorbance at 490nm was measured with CliniBio 128 (ASYS-Hitech, Austria). The half maximal inhibitory concentration (IC50) was calculated using probit analysis with SPSS 22.0 (SPSS Inc, Chicago, US).

### Colony formation assay

MDA-MB-231/S cells were transfected with mimics or the negative control of mimics (NC) and placed onto a fresh plate at a density of 200 cells. After 24 hours of incubation, cells were treated with 2mL of Epirubicin(0.364uM). Then, cells were cultured for an additional 10 days, and then stained with Giemsa. The experiments were performed for three times.

### Apoptosis assay

Transfected MDA-MB-231/S or MDA-MB-231/Epi cells(5×10^5^) were seeded in each well of six-well plates for 24 h, following adding 0.1mM Epi to each plate for 24h. Then, the cells were stained with 5μl of APC Annexin V and 5ul of 7-AAD (BD Pharmingen, America) in the dark for 15 min. At last, each tube was added 400ul of 1X binding buffer and analyzed with the flow cytometer (FACSVerse/Calibur/AriaII-SORP, BD, America). Three replications of each experiment were performed.

### Bioinformatic analysis

The miR-4443 target gene—TIMP2 was predicted using TargetScan software (http://www.targetscan.org/). Further correlation between patient survival and TIMP2 expression was determined through analysis of The Cancer Genome Atlas(http://cancergenome.nih.gov/).

### Western blot

Total proteins were collected from cells 24h after transfection following harvested using a RIPA lysis buffer(Biouniquer Technology, Nanjing, China) based on the manufacturer’s instruction. Then the purity and quality of proteins were measured with Nanodrop 2000 spectrophotometry (Thermo Scientific, USA). Equal amounts of proteins were separated by electrophoresis using 8% sodium dodecyl sulfate (SDS) polyacrylamide gels before transferring to polyvinylidene difluoride membranes (Sigma, Germany). The membranes were blocked in 5% skim milk for 2 h and then probed with primary antibodies against TIMP2 (1:250; Biotechne, America) or β-actin (1:8000, Arigo, China) at 4°C overnight, followed by the secondary antibody (1:4000; Kangwei Ltd, China) for 1 h. Finally, after washing, enhanced chemiluminescence (ECL) plus kit (Millipore, America) was applied for visualization and β-actin was utilized as an internal control.

### Statistical analysis

All statistical analyses were performed using Stata13.1, SPSS 22.0 and GraphPad Prism5. The comparison of miRNA expression level in paired tissue blocks of individual patient was performed using Wilcoxon matched pairs signed rank test. Wilcoxon-Mann-Whitney rank sum test was applied to assess miRNA expression differences in SD/PD group and PR group. The χ2 test or Fisher exact test(when total cases were less than 40 or absolute value was smaller than 5) was used to analyze the association of miR-4443 expression level with clinicopathological characteristics. The data presented on FFPE tissues were one-sided, and a p-value<0.05 was considered to be statistically significant. Besides, the data were presented as mean ± standard error with the representative images. One-way analysis of variance (ANOVA) followed by the Student–Newman–Keuls post-hoc test was used to assess the statistical significance of difference between cell groups. Two-sided p-values<0.05 on cells was considered statistically significant.

## Results

### Identification of miR-4443 expression in MDA-MB-231 cells

**Fig 1:**
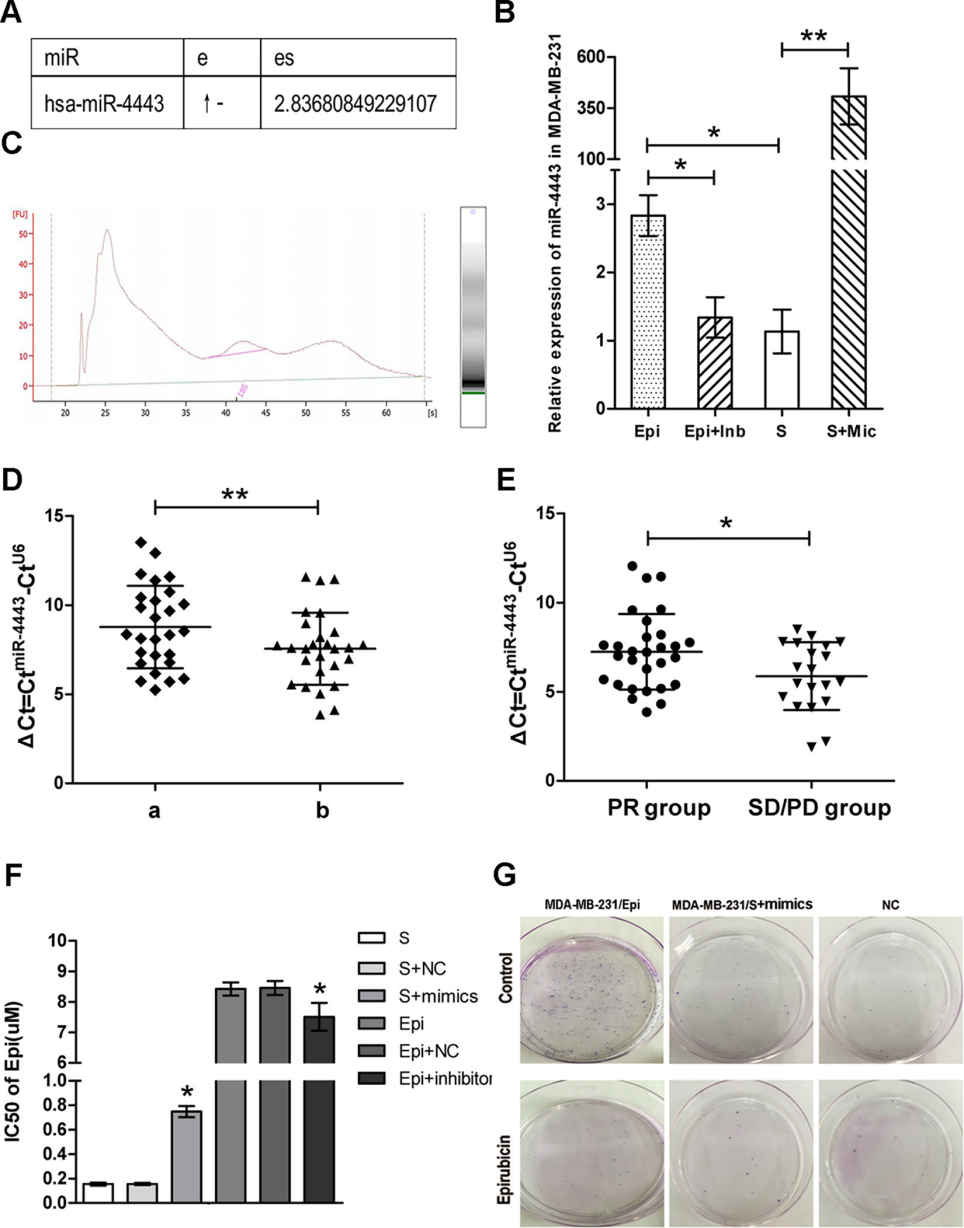
Association of miR-4443 expression and drug resistance. **(A)**, Microarray result of miR-4443 in drug resistant breast cancer cell lines. e: epiadriamycin- resistant MDA-MB-231 cell line; es: expression status of miRNA in epiadriamycin- resistant MDA-MB-231 cell line. **(B)**, Quantification of miR-4443 in MDA-MB-231/Epi and MDA-MB-231/S cell lines and with miR-4443 Inhibitors or Mimics respectively(p<0.05). Epi+Inb: MDA-MB-231/Epi transfected with miR-4443 Inhibitors; S+Mic: MDA-MB-231/S transfected with miR-4443 mimics. **(C)**, Quality of extracted RNA. **(D)**, Quantification of miR-4443 in a)pre-neoadjuvant chemotherapy FFPE biopsies and b)surgically-resected FFPE specimens of 27 patients(p<0.001). **(E)**, Quantification of miR-4443 in PR and SD/PD groups(p<0.05). ΔCt values for miRNA studied are shown referenced to the expression of the endogenous control, U6. PR: partial response, SD: stable disease, PD: progressive disease. **(F)**, IC50 value of targeted cells transfected with miR-4443 mimics or inhibitors. Epi+inhibitors: MDA-MB-231/Epi transfected with miR-4443 Inhibitors; S+mimics: MDA-MB-231/S transfected with miR-4443 mimics; NC: negative control of miR-4443 mimics or inhibitors. **(G)**, The colony formation assay in MDA-MB-231/S.

The miRNA expressions of MDA-MB-231/Epi and MDA-MB-231/S were evaluated by using the Affymetrix GeneChip miRNA 4.0 Array. Compared with MDA-MB-231/S cell line, the expression level of miR-4443 was upregulated approximately 2.83-fold in the MDA-MB-231/Epi cell line(Fig 1A). Therefore, we evaluated the expression of miR-4443 in MDA-MB-231/Epi and MDA-MB-231/S cells for RT-qPCR validation, which showed a good consistency with the microarray results (Fig 1B, p<0.05).

### MiR-4443 expression in patient FFPE samples

The characteristics of the patients are presented in Table 1. The quality of extracted RNA was shown in Fig 1C. We detected the miR-4443 expression in 27 paired FFPE specimens to explore whether miR-4443 was associated with drug resistance *in vivo*. Our results showed that the expression level of miR-4443 was significantly up-regulated after neoadjuvant chemotherapy(ΔCt = 7.55±2.02) compared to those in biopsy specimens(ΔCt = 8.76±2.31)(Fig 1D, p<0.001). When compared with miRNA expression in the PR group(ΔCt = 7.23±2.12), miR-4443 in surgically-resected specimens was significantly up-regulated in SD/PD group(ΔCt = 5.87±1.90) (Fig 1E, p<0.05), while no significant difference was found in chemotherapy-free biopsy FFPE specimens(S1 Fig, p>0.05). Besides, we selected the median of the miR-4443 relative expression(ΔCt = 6.91) as cut-off value and after analysis we found that miR-4443 had no significant differences in age, chemotherapy regimens, tumor stage, lymph nodes stage, estrogen receptor status, progesterone receptor status and Her-2 status of breast cancer patients.

**Table 1:** Clinicopathological characteristics and expression of miR-4443 in breast cancer.

Characteristics		miR-4443		
Age(years)	cases	low	high	p
≤55	23	11	12	0.882
>55	24	12	12	
Tumor stage				
I	10	4	6	0.578
II	32	16	16	
III	4	3	1	
N stage				
0	27	11	16	0.155
1–4	9	7	2	
>4	11	5	6	
ER status				
positive	35	17	18	0.936
negative	10	5	5	
PR status				
positive	34	16	18	0.677
negative	13	7	6	
Her-2 status				
positive	40	18	22	0.245
negative	7	5	2	
Regimen				
TAC	25	10	15	0.252
TC	23	13	10	

TAC: Taxel, Adriamycin, Cyclophosphamide; TC: Taxel, Cyclophosphamide; ER: estrogen receptor; PR: progesterone receptor; Her-2: human epidermal growth factor receptor.

### MiR-4443 mimics or inhibitors partially changed the drug-resistance of breast cancer cells

The miR-4443 mimics were transfected into MDA-MB-231/S cells to explore whether the transfected cells would acquire anticancer drug resistance. RT-qPCR analysis indicated that miR-4443 was up-regulated by 407.56-fold compared to negative control of mimics (NC) in MDA-MB-231/S cells(Fig 1B, p<0.001). The result of MTT assay showed that the half maximal inhibitory concentration(IC50) value of Epirubicin for the target cells transfected by miR-4443 mimics significantly increased, compared with those of the control cells (Fig 1F). The colony formation assay performed similar results. The clonal survival in MDA-MB-231/S transfected with mimics greatly increased (Fig 1G).

Simultaneously, miR-4443 inhibitors were transfected into MDA-MB-231/Epi sublines. The result of RT-qPCR analysis showed that miR-4443 was down-regulated by 0.47-fold compared to negative control of inhibitors (NC) in MDA-MB-231/Epi cells(Fig 1B, p<0.05). The MTT assay results demonstrated that miR-4443 inhibitors could restore sensitivity of the target cells to Epi in some degree (Fig 1F).

### Involvement of miR-4443 in Epi-induced apoptosis

**Fig 2:**
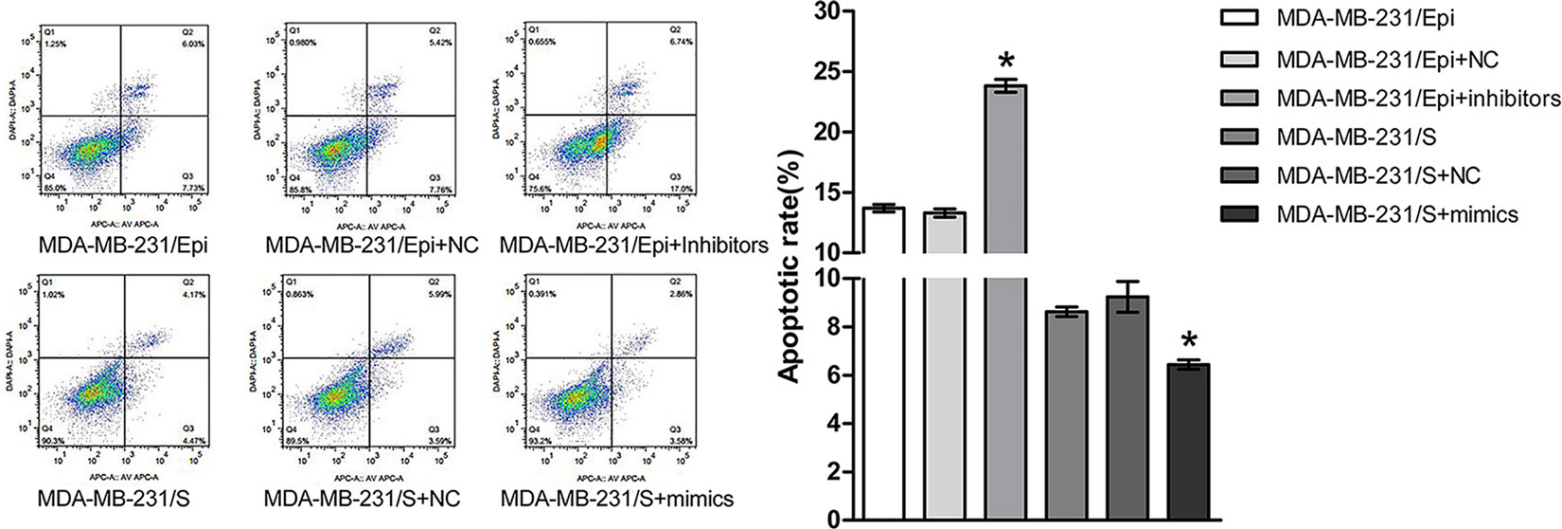
Apoptotic rates of cells transfected with miR-4443 mimics or inhibitors (p<0.05). MDA-MB-231/Epi+inhibitors: MDA-MB-231/Epi transfected with miR-4443 Inhibitors; MDA-MB-231/S+mimics: MDA-MB-231/S transfected with miR-4443 mimics; NC: negative control of miR-4443 mimics or inhibitors.

Apoptosis assay revealed that down-regulation of endogenous miR-4443 significantly enhanced Epi-induced apoptosis in MDA-MB-231/Epi cells compared to negative controls (Fig 2, p<0.01).

Besides, when transfected with miR-4443 mimics, MDA-MB-231/S cells presented prominently less Epi-induced apoptotic cells by apoptosis assay, compared to negative controls(Fig 2, p<0.01).

### MiR-4443 regulate expression of TIMP2

**Fig 3:**
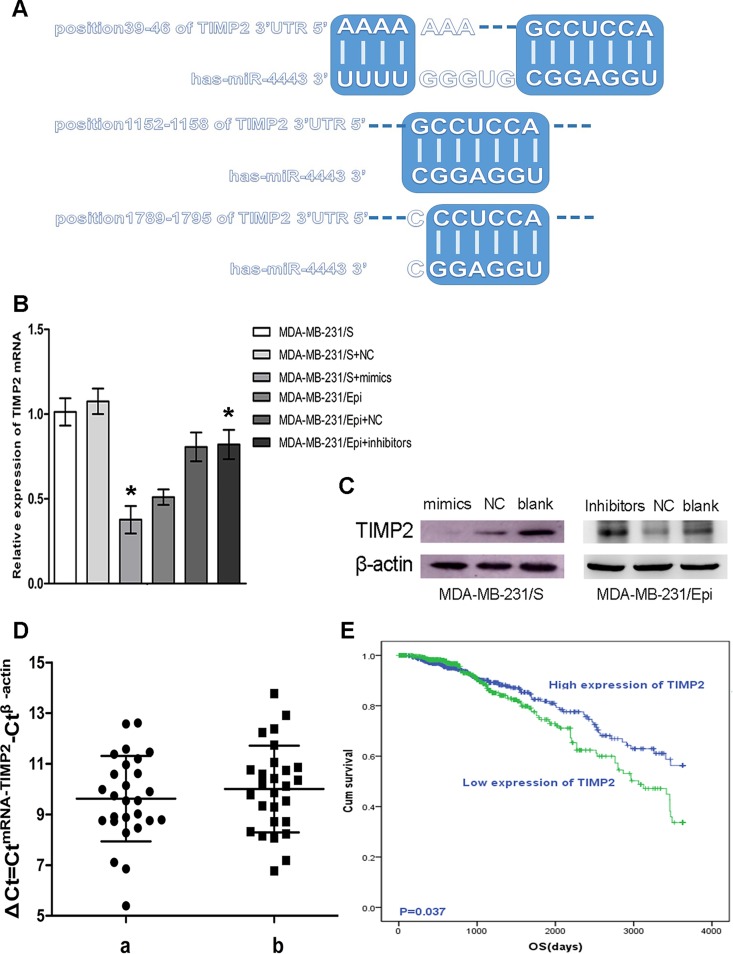
Relationship between TIMP2 and miR-4443 with survival. **(A)**, Interactions between TIMP2 and miR-4443 by TargetScan software. **(B)**, Quantification of TIMP2 mRNA mediated by miR-4443 in MDA-MB-231 cells. ΔCt values for mRNA are shown referenced to the expression of the endogenous control, β-actin. MDA-MB-231/Epi+inhibitors: MDA-MB-231/Epi transfected with miR-4443 Inhibitors; MDA-MB-231/S+mimics: MDA-MB-231/S transfected with miR-4443 mimics; NC: negative control of miR-4443 mimics or inhibitors. **(C)**, Relative protein expression of TIMP2, β-actin is the endogenous control. **(D)**, Quantification of TIMP2 mRNA in a)pre-neoadjuvant chemotherapy FFPE biopsies and b)surgically-resected FFPE specimens of 27 patients(p = 0.061). **(E)**, Survival curves of TIMP2(p<0.05).

The results of TargetScan software identified 3 complementary binding sites of miR-4443 in TIMP2 3’ UTR including positions of 39–46, 1152–1158, 1789–1795 (Fig 3A). In order to investigate whether miR-4443 could down-regulate TIMP2 expression, the corresponding mimics and inhibitors of the miRNA were transfected into MDA-MB-231/S and MDA-MB-231/Epi separately. TIMP2 mRNA expression was determined at 24 h after transfection. The results showed that TIMP2 mRNA expression in MDA-MB-231/S cells transfected with miR-4443 mimics was significantly decreased compared with that in MDA-MB-231/S cells transfected with negative controls, and the TIMP2 mRNA expression in MDA-MB-231/Epi cells transfected with miR-4443 inhibitors was significantly increased compared with that in MDA-MB-231/Epi cells transfected with negative controls (Fig 3B; p<0.05). Simultaneously, after transfecting miR-4443 mimics, TIMP2 protein expression in MDA-MB-231/S cells was evidently elevated comparing to negative controls, and depression of miR-4443 by inhibitors was related to up-regulation of TIMP2 protein expression in contrast with negative controls. (Fig 3C; p<0.05). Meanwhile, mRNA of TIMP2 in FFPE tissues delivered higher relative expression in biopsy specimens than paired postoperative specimens of 27 breast cancer patients(Fig 3D), the difference of ΔCt in paired specimens was 0.38±1.32. However, no statistically significant differences were found(p = 0.061).

### TIMP2 expression correlates with survival

To further evaluate the potential relationship of TIMP2 expression with patient survival, we generated Kaplan-Meier survival curves from TCGA dataset. The results demonstrated that inhibition of TIMP2 expression informed poorer prognosis(Fig 3E; HR = 0.721, 95%CI: 0.529–0.983, p<0.05).

## Discussion

The present study may give early warning of a unique biomarker in malignancy of breast cancer patients. The precious results depicted that miR-4443 was involved in the acquired drug resistance of breast cancer *in vitro* and *in vivo* for the first time, therefore providing the potential biomarker with therapeutic targets for guiding effective treatments and ensuring the overall success of breast cancer therapies.

Chemotherapy is an effective treatment which restrains tumor growth and controls many primary tumors. However, the drug-resistant acquisition in cancer cells remains serious, thus current cancer treatments rarely stop the metastatic spread[15]. MiRNAs are considered as novel biomarkers and therapeutic targets for cancer resulting from their involvements in cancer initiation and progression, as well as chemo-resistance[16].

Increasing evidences have shown that chemo-resistance in currently used treatments might be correlated to dysregulation of miRNAs via interfering protein expression within cells, the ability for anti-cancer drugs to bind to their targets, or the apoptotic pathways[17]. And controlling the expression of aberrantly expressed miRNAs has been proved to transfer resistant phenotype of breast cancer cells to nonresistant ones and to bring about successful therapeutic outcomes in breast cancer cells[18–22]. MiR-4443 was once detected to be associated with drug resistance[23]. These findings are similar with our results that miR-4443 was upregulated in drug resistant cells(MDA-MB-231/Epi) compared with MDA-MB-231/S cells which was consistent with the microarray data *in vitro*. Also, the expression level of miR-4443 in postoperatively surgical-resected FFPE tissues after neoadjuvant chemotherapy was markedly higher than those paired needle biopsy tissues before treatment in breast cancer patients, which indicating that miR-4443 induced acquired drug resistance *in vivo*. Besides, miR-4443 in surgically-resected specimens was significantly up-regulated in SD/PD group compared with that in PR group, which emphasized the role of miR-4443 in chemotherapy. Mechanically, miR-4443 inhibitors could restored sensitivity of the target cells to Epi in some degree according to the elevated apoptotic rate and decreased IC50 value, along with the attenuated sensitivity of the mimic-transfected cells to chemotherapeutic drugs. These results showed that miR-4443 could confer Epi resistance both *in vitro* and *in vivo*. Simultaneously, miR-4443 mimics induced MDA-MB-231/S cells to exhibit prominently less Epi-induced apoptotic rate compared to negative controls. Also, inhibition of miR-4443 expression in MDA-MB-231/Epi cells by miRNA inhibitors led to a greater Epi-induced apoptotic rate compared to negative controls. The results strengthen the resistant role of miR-4443 in MDA-MB-231 breast cancer cells. For the reason that we don’t have another Epi-resistant cell line, so we chose the MCF-7/S and MCF-7/Adr(MCF-7 Adriamycin resistant) cells for Epirubicin and Adriamycin are both Anthracycline. Compared with MCF-7/S cell line, the expression level of miR-4443 was upregulated approximately 0.87-fold in the MCF-7/Adr cell line. When transfected with miR-4443 mimics, MCF-7/S cells presented prominently less Adr-induced apoptotic cells by apoptosis assay, compared to negative controls(S2 Fig, p<0.05). The results confirmed that miR-4443 related to drug resistance in MCF-7 cell lines, although further studies were expected.

According to the results of TargetScan analysis, we found TIMP2, as natural inhibitors of MMP-2[24], was one of the target genes of miR-4443, which was partly verified by the imitation or inhibition of TIMP2 mRNA expression in mimics-transfected MDA-MB-231/S or inhibitors-transfected MDA-MB-231/Epi cells compared with the negative controls *in vitro*. Relative expressions of TIMP2 mRNA in postoperatively surgical-resected FFPE tissues after neoadjuvant chemotherapy were lower than those in the paired needle biopsy tissues before treatment in breast cancer patients, which also testified that miR-4443 was targeted to TIMP2 in the pre-transcriptional association. However, no statistically significant difference was found in the FFPE tissues of breast cancer patients, and this may have resulted from the potential role of TIMP2 in malignancy of breast cancer or the small number of available patients. We also found that TIMP2 was associated with better survival rate of breast cancer patients. Therefore, miR-4443 probably added to the chemoresistant capability of breast cancer cells by targeting TIMP2.

In conclusion, this study demonstrated that overexpression of miR-4443 was involved in the malignancy of breast cancer both *in vitro* and *in vivo*, which suggested that specified miRNAs could serve as precious sources for the biomarker detection and optimal chemotherapeutic choice for breast cancer patients.

## Supporting Information

S1 Fig Quantification of miR-4443 in PR and SD/PD groups in 27 biopsy FFPE tissues(p>0.05). ΔCt values for miRNA studied are shown referenced to the expression of the endogenous control, U6. PR: partial response, SD: stable disease, PD: progressive disease
(TIF)

S2 Fig Association of miR-4443 expression and drug resistance in MCF-7 cell line. **(A)**, Quantification of miR-4443 in MCF-7/Adr and MCF-7/S cell lines(p<0.05). **(B)**, Apoptotic rates of cells transfected with miR-4443 mimics(p<0.05). MCF-7/S+mimics: MCF-7/S transfected with miR-4443 mimics; NC: negative control of miR-4443 mimics.
(TIF)
