# Supplementary figures and images for: miR-4443 Participates in the Malignancy of Breast Cancer
Source: PLoS One. 2016 Aug 9;11(8):e0160780. doi: 10.1371/journal.pone.0160780 (PMC4978484; doi:10.1371/journal.pone.0160780)

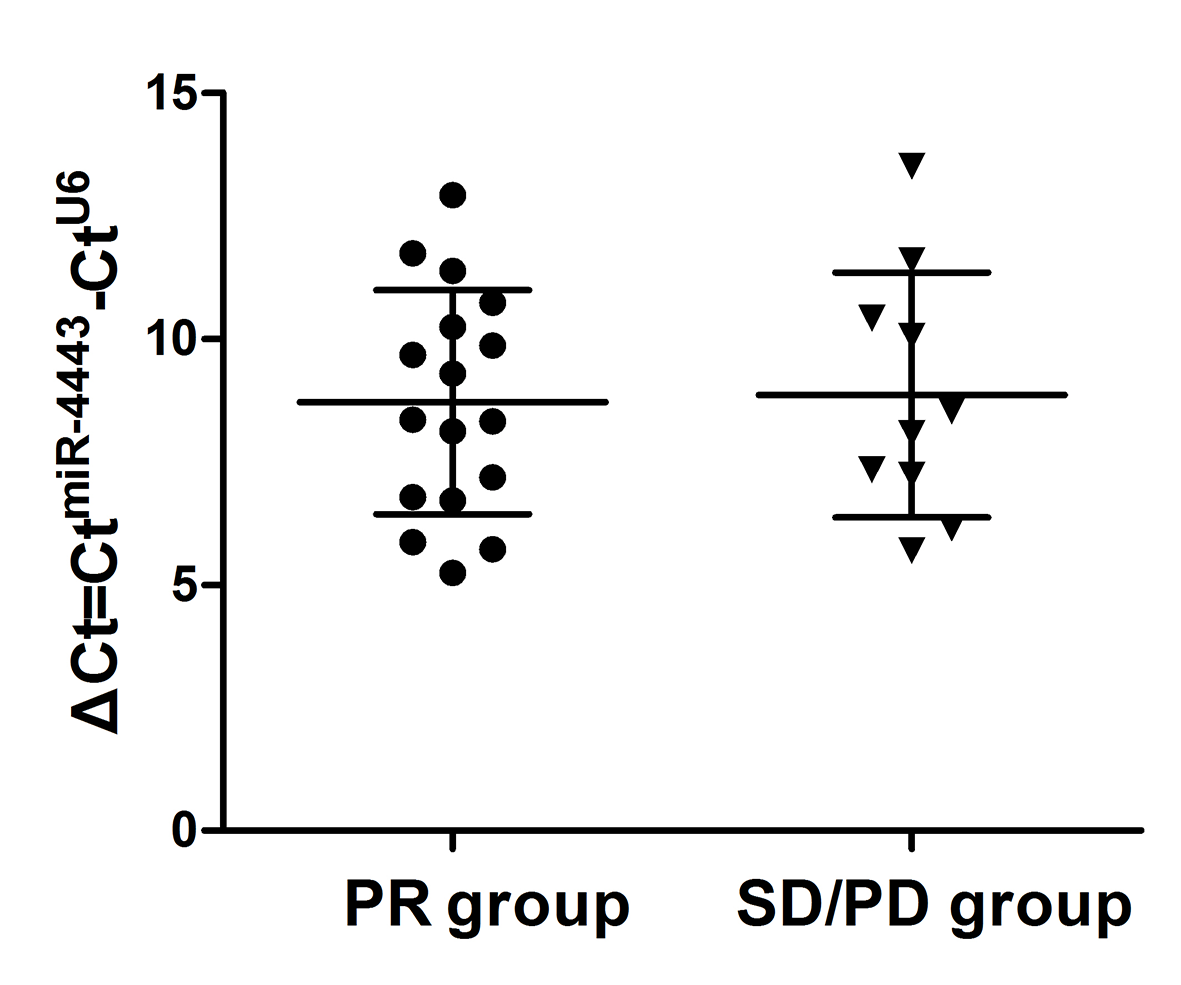

Supplement: S1 Fig — ΔCt values for miRNA studied are shown referenced to the expression of the endogenous control, U6. PR: partial response, SD: stable disease, PD: progressive disease (TIF) [file pone.0160780.s001.tif]

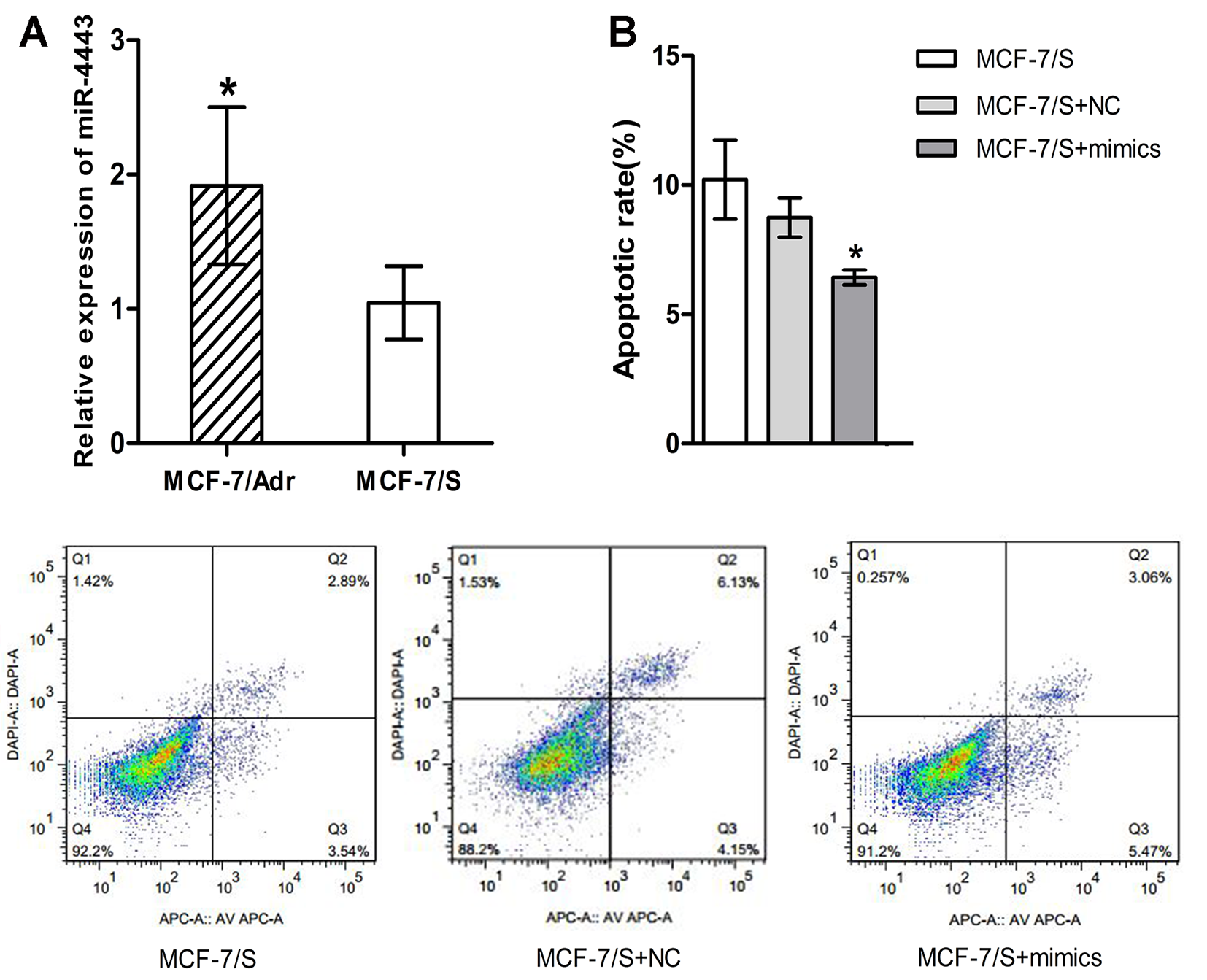

Supplement: S2 Fig — (A), Quantification of miR-4443 in MCF-7/Adr and MCF-7/S cell lines(p<0.05). (B), Apoptotic rates of cells transfected with miR-4443 mimics(p<0.05). MCF-7/S+mimics: MCF-7/S transfected with miR-4443 mimics; NC: negative control of miR-4443 mimics. (TIF) [file pone.0160780.s002.tif]
